# Assessment of Clinical Factors that Predict Response to Nabilone for Agitation in Alzheimer's Disease: A Post Hoc Analysis of a Randomized Control Trial

**DOI:** 10.1002/alz70857_103582

**Published:** 2025-12-25

**Authors:** Oriel J Feldman, Myuri Ruthirakuhan, Nathan Herrmann, Damien Gallagher, Giovanni Marotta, Alex Kiss, Hui Jue (Janet) Wang, Yejin Kang, Sandra E. Black, Krista L Lanctôt

**Affiliations:** ^1^ University of Toronto, Toronto, ON, Canada; ^2^ Sunnybrook Research Institute, Toronto, ON, Canada; ^3^ Temerty Faculty of Medicine, University of Toronto, Toronto, ON, Canada; ^4^ Sunnybrook Health Sciences Centre, Toronto, ON, Canada; ^5^ Neuropsychopharmacology Research Group, Sunnybrook Research Institute, Toronto, ON, Canada; ^6^ Department of Psychiatry, Temerty Faculty of Medicine, University of Toronto, Toronto, ON, Canada; ^7^ Hurvitz Brain Sciences Program, Sunnybrook Research Institute, Toronto, ON, Canada; ^8^ Geriatric Psychopharmacology Research Group, Sunnybrook Research Institute, Toronto, ON, Canada; ^9^ Hurvitz Brain Sciences Program, Toronto, ON, Canada; ^10^ Division of Neurology, Department of Medicine, University of Toronto, Toronto, ON, Canada

## Abstract

**Background:**

Agitation is one of the most prevalent neuropsychiatric symptoms (NPS) of Alzheimer's disease (AD). A crossover trial of nabilone for agitation in AD found that nabilone was effective in treating agitation. We aimed to identify which clinical characteristics predicted response to nabilone intervention for agitation.

**Method:**

Twenty‐two potential clinical characteristics were identified *a priori*. Characteristics were analyzed for their ability to predict improvements on the Cohen‐Mansfield Agitation Inventory (CMAI). Each characteristic was categorically split and compared with univariate analyses: characteristics showing differences ≥8 CMAI points were included in a multivariate regression to model interactions between the treatment and characteristics. Index scores were calculated to represent the likelihood of response to treatment and results were grouped into quartiles. Multicollinearity of the characteristics was assessed.

**Result:**

35 patients (28 males (80%), mean age [SD] 87.0 [10.2] years, CMAI 67.4 [17.7], standardized Mini‐Mental State Exam (sMMSE) 6.6 [6.8]) had complete data for clinical predictors, allowing for 70 cases to be analyzed. Six predictors met criteria for inclusion in multivariate modelling, where nabilone was more effective in participants with sMMSE scores ≥6 (Δ level estimates = ‐12.0), higher levels of pain (‐17.1), apathy (‐8.7), appetite and eating changes (‐9.7), and not taking cholinesterase inhibitors (ChEIs) (‐8.4).

**Conclusion:**

AD patients with pain, apathy and appetite changes, and with less cognitive impairment and not on ChEIs, were most likely to benefit from nabilone. If replicated with phase 3 data, these predictors may assist in guiding clinicians on who is likely to benefit from nabilone when managing agitation.

